# Validation of a surgical model for posthepatectomy liver failure in rats

**DOI:** 10.1002/ame2.12325

**Published:** 2023-05-14

**Authors:** Andrea Lund, Michelle Meier, Kasper Jarlhelt Andersen, Marie Ingemann Pedersen, Anders Riegels Knudsen, Jakob Kirkegård, Frank Viborg Mortensen

**Affiliations:** ^1^ Aarhus University Hospital Department of Upper Gastrointestinal and Hepato‐Pancreato‐Biliary Surgery Aarhus N Denmark

**Keywords:** 90% liver resection, general distress score, liver failure, post‐hepatectomy liver failure, rats

## Abstract

**Background:**

The upper limit for liver resections in rats is approximately 90%. In the early postoperative phase, mortality increases. The aim of the present study was to validate the rat model of 90% partial hepatectomy (PH) as a model of post‐hepatectomy liver failure (PHLF). Further, we wanted to test a quantitative scoring system as a detector of lethal outcomes caused by PHLF in rats.

**Methods:**

Sixty‐eight rats were randomized to 90% PH, sham operation, or no surgery. Further, block randomization was performed based on time of euthanization: 12, 24, or 48 h after surgery. A general distress score (GDS) ≥10 during the day or ≥6 at midnight prompted early euthanization and classification as nonsurvivor. Animals euthanized as planned were classified as survivors. During euthanization, blood and liver tissue were collected, and liver‐specific biochemistry was evaluated.

**Results:**

Based on the biochemical results, all animals subjected to 90% PH experienced PHLF. Seventeen rats were euthanized due to irreversible PHLF. The GDS increased for nonsurvivors within 12–18 h after surgery. The mean time for euthanization was 27 h after surgery.

**Conclusion:**

Based on the GDS and liver‐specific biochemistry, we concluded that the model of 90% PH seems to be a proper model for investigating PHLF in rats. As a high GDS is associated with increased mortality, the GDS appears to be valuable in detecting lethal outcomes caused by PHLF in rats.

## Graphical abstract

In the present study, we evaluated the rat model of 90% partial hepatectomy (PH) as a model of post‐hepatectomy liver failure (PHLF). We also investigated the use of a quantitative scoring system to detect lethal outcomes caused by PHLF in rats. Based on our clinical scoring system and supported by liver‐specific biochemistry, the model of 90% PH seems to be relevant for investigating PHLF in rats. By including both physiological and behavioral parameters, GDS appears to be valuable in detecting lethal outcomes caused by PHLF in rats.

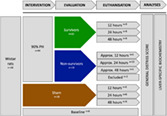

## 1 INTRODUCTION

Liver malignancy is a major health‐care burden globally, and the incidence of both primary and secondary liver malignancies is increasing.^1^, ^2^ Resection of the tumor, that is, partial hepatectomy (PH), represents the gold standard of treatment and is facilitated by the liver's unique ability to regenerate. In healthy humans, the future liver remnant (FLR) should be at least 25%–30% of the initial liver volume to maintain vital liver functions.^3^, ^4^ A volume below this limit increases the risk of post‐hepatectomy liver failure (PHLF), which is the main reason for postoperative short‐term mortality after extended PH.^5^, ^6^, ^7^

The multilobular structure of the rat liver makes the rat an ideal animal to use in surgical liver models.^8^, ^9^ Madrahimov et al. developed a three‐dimensional model of the hepatic vascular anatomy in rats, presenting the relative amount of each liver lobe and its blood supply.^10^ The left lateral lobe (LLL) represents 30% of the total liver volume, the median lobe (ML) 40%, the right superior lobe (RSL) 13%, the right inferior lobe (RIL) 6%, the caudate lobes 7%, and the paracaval liver tissue 3%. Based on this anatomical knowledge, PH varying from 30% to 95% has been widely used for investigating liver regeneration in rats, suggesting that only 5%–10% of the initial liver volume is needed to maintain vital functions for survival.^9^, ^10^, ^11^

Investigating the pathophysiology involved in PHLF is essential to guiding intervention efforts to avoid and treat PHLF, thereby improving outcomes after extensive PH. The experimental rat model of 90% PH might be a useful model of PHLF, as it seems to mimic the critical regenerative phase of the minimal‐size FLR,^12^ in which biliary excretion is decreased, the liver synthetic function is impaired, and mortality is increased.^12^, ^13^, ^14^ Previous studies on rats have indicated that irreversible PHLF generally occurs within the first 72 h after surgery,^12^, ^13^ and 70% of the lethal outcomes occur within 24 h.^13^

Unfortunately, the presence of severe PHLF and fatal progression can be difficult to assess in rats, because continuously collecting blood samples is not feasible due to the small blood volume. Therefore, to investigate liver regeneration and function in rats suffering from PHLF, it is essential to explore another method for monitoring PHLF. Behavioral scoring systems used to assess animal pain and suffering could be a helpful tool for this purpose. One commonly used method is the general distress score (GDS), which is a quantitative scoring system of physiological and behavioral parameters that allows the implementation of ethically acceptable endpoints.^15^

The aim of the present study was to validate the rat model of 90% PH as a model of PHLF. In addition, we aimed to evaluate GDS as a quantitative scoring system for detecting lethal outcomes caused by PHLF in rats.

## 2 METHODS

### 2.1 Animals

Healthy, 7‐week‐old male Wistar rats (Janvier Labs, Le Genest‐Saint‐Isle, France) with a mean preoperative weight of 270 g (range: 240–300 g) were housed in standard animal laboratories with a temperature maintained at 23°C, an artificial 12‐h light–dark cycle, and free access to food and water. The rats were kept in these animal facilities until the end of the experiment.

### 2.2 Experimental design

#### 2.2.1 Preliminary study

First, we conducted a pilot study in which the primary investigator performed 90% PH in 60 male Wistar rats. This was performed to train the investigator in the procedure, ultimately reducing intra‐operator variability and standardizing the procedure. Further, different microsurgical strategies and perioperative regimes were tested to obtain a high reproducibility of the procedure.

#### 2.2.2 Main study

**FIGURE 1:**
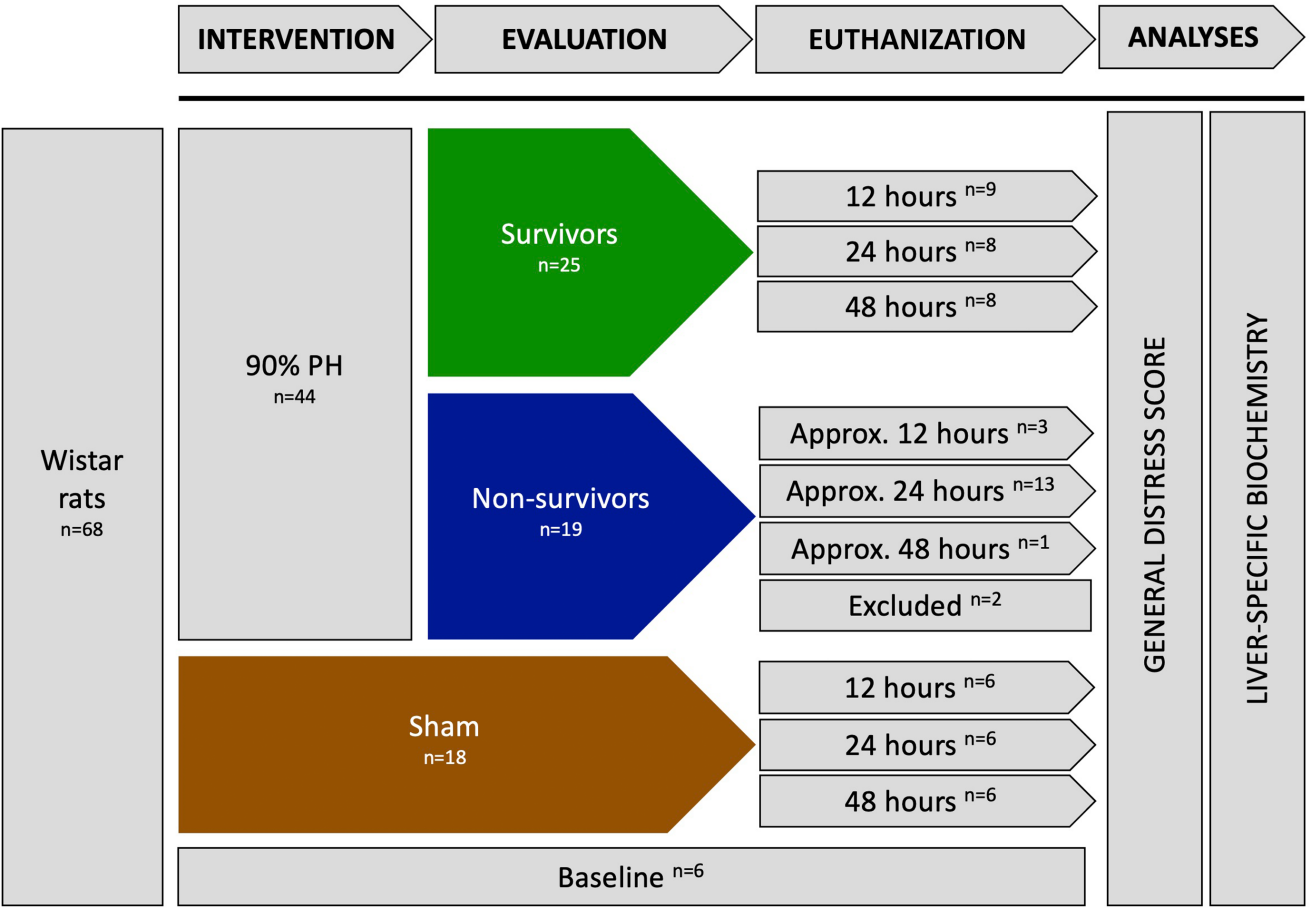
Study design including 68 male Wistar rats randomized based on intervention and time of euthanization. The GDS (general distress score) was used to categorize animals as survivors or nonsurvivors. Two animals were excluded in the analyses: one died unexpectedly and one due to vena cava compression.

Sixty‐eight male Wistar rats were randomized into blocks of two to either intervention, that is, 90% PH (*n* = 44), or a sham‐group, with animals undergoing laparotomy without liver resection (*n* = 18) (Figure 1). Each group was further block randomized into three subgroups to euthanization at 12 h (*n* = 10), 24 h (*n* = 14), or 48 h (*n* = 20) after surgery. In addition, we included a baseline group (*n* = 6) with animals not exposed to surgery.

Animals presenting with a GDS ≥10 during the day or a GDS ≥6 at midnight were euthanized. Animals euthanized before planned evaluation were allocated to the “nonsurvivors” group and to the subgroup closest to the time of euthanization. Animals alive at the planned time of euthanization were allocated to the “survivors” group.

### 2.3 Surgical procedure

All animals were weighed before receiving a subcutaneous injection of 150 mg/kg of ampicillin (STADA Nordic), 5 mg/kg of carprofen (Rimadyl Bovis vet, Zoetis), and 2.0 mL of isotonic saline.

The surgical procedures were performed under inhalation anesthesia: induction was performed in a glass cylinder with a mixture of oxygen (0.3 L/min), nitrous oxide (0.15 L/min), and sevoflurane (5%) (Baxter Medical, Kista, Sweden). During surgery, anesthesia was maintained using 3.5% sevoflurane, oxygen, and nitrous oxide administered by a mask covering the animals' mouth and nose.

**FIGURE 2:**
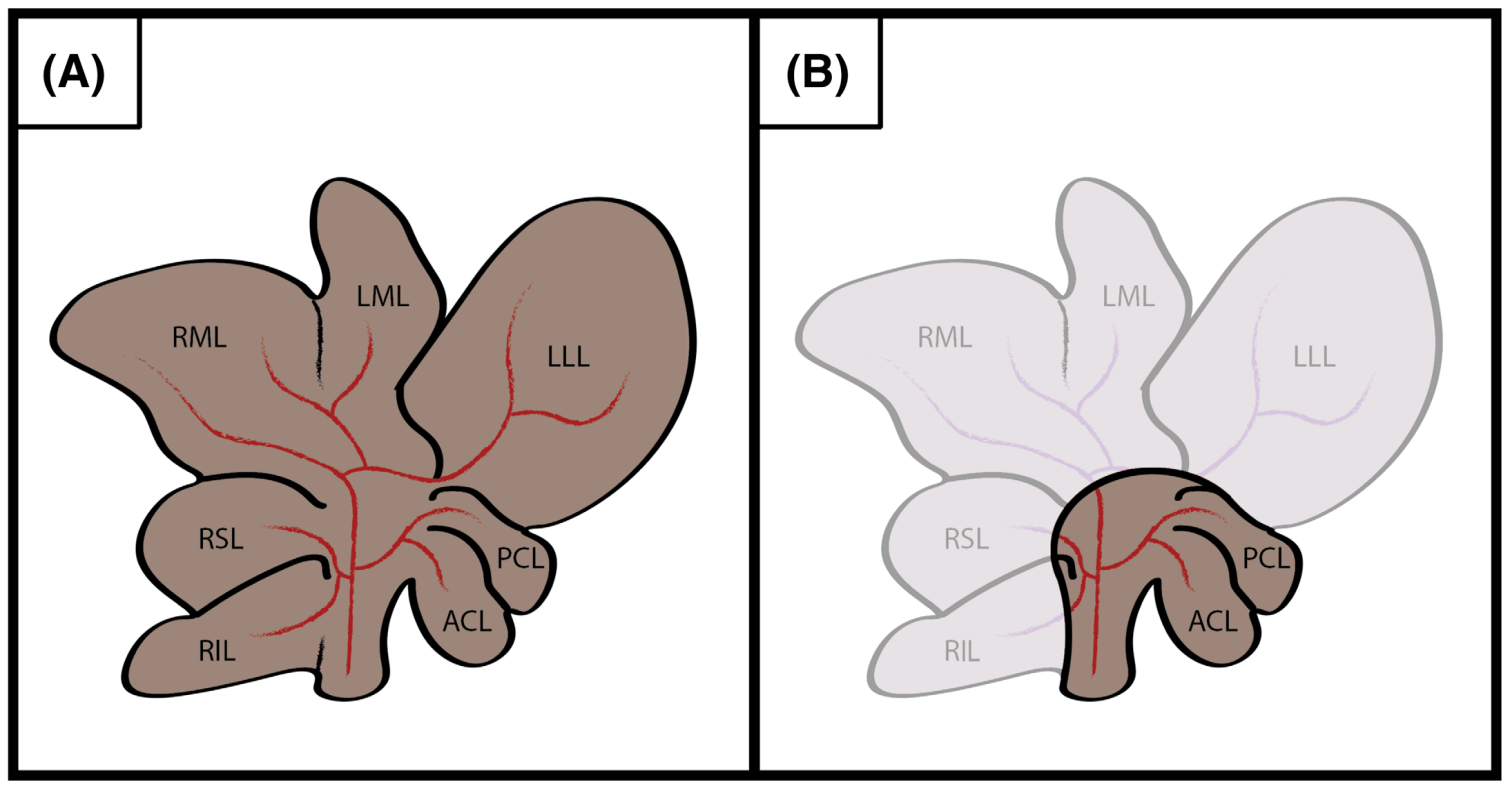
(A) Normal rat liver anatomy. (B) The liver remnant after 90% PH (partial hepatectomy), consisting of ACL and PCL. ACL, anterior caudate lobe; LLL, left lateral lobe; LML, left median lobe; PCL, posterior caudate lobe; RML, right median lobe; RIL, right inferior lobe; RSL, right superior lobe.

**FIGURE 3:**
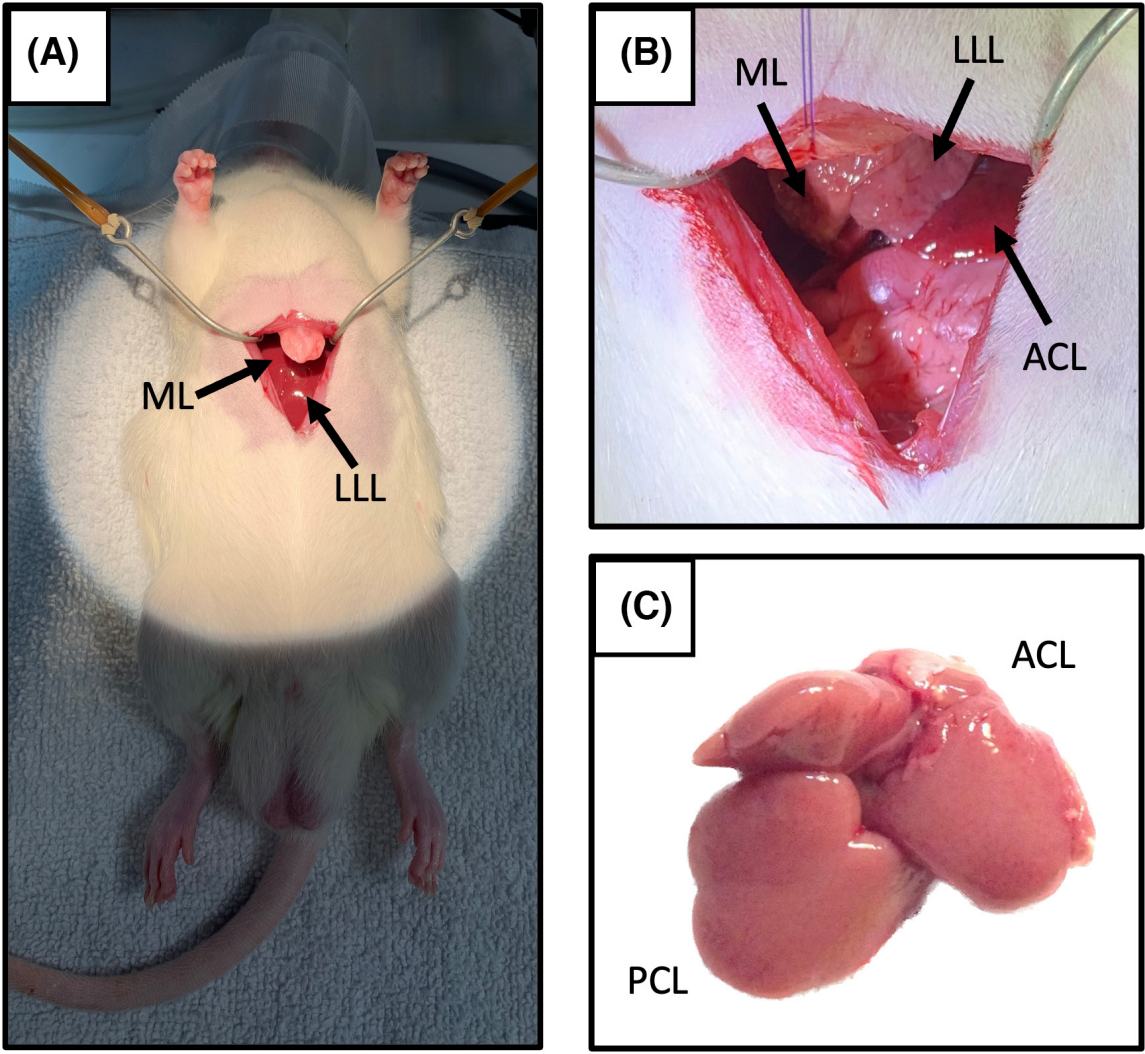
(A) Opening of the abdomen. From the outside, the liver is the only visible organ. (B) To get a better overview, the xiphoid process was carefully lifted. The four liver lobes were mobilized and resected, leaving only the two caudate lobes intact. The PCL (posterior caudate lobe) is found behind the ACL (anterior caudate lobe). Resection margins of the ML (median lobe) and the LLL (left lateral lobe) are observed cranially and centrally to the ACL. (C) The liver remnant collected 48 h after 90% PH (partial hepatectomy). Both caudate lobes have increased markedly in size.

With the animal placed in supine position on a heated pad, a 3‐cm midline incision was made (Figures 2 and 3). Mobilization of the liver was performed by removing three ligaments: the falciform ligament, the interlobular ligament between the anterior caudate lobe (ACL) and LLL, and the left triangular ligament attaching LLL to the diaphragm. RSL, RIL, ML, and LLL were ligated and resected, leaving only the ACL and posterior caudate lobe (PCL) intact. The liver pedicles were ligated using a 5‐0 reabsorbable monofilament, and liver tissue was transected using a dissecting scissor. Because of the vascular anatomy, care was taken to place the ligature at a distance of 3 mm to the caval vein when ligating ML and RSL.^10^, ^11^

After liver resection, the liver remnant was inspected to detect potential ischemia, torsion of the pedicles, or compression of the vena cava before closing the abdomen. A well‐perfused liver remnant was found to be light red. Ischemia was expressed by white color, and the liver remnant turned red and swollen if stasis was present due to vena cava compression. In case of liver ischemia or stasis (*n* = 1), animals were excluded from the study. The resected liver tissue was weighed. The abdomen was closed intramuscularly using a 4‐0 reabsorbable suture, and the skin was closed with 7.5‐mm staples (Agraf Michel, Medicon).

### 2.4 Clinical observations

**FIGURE 4:**
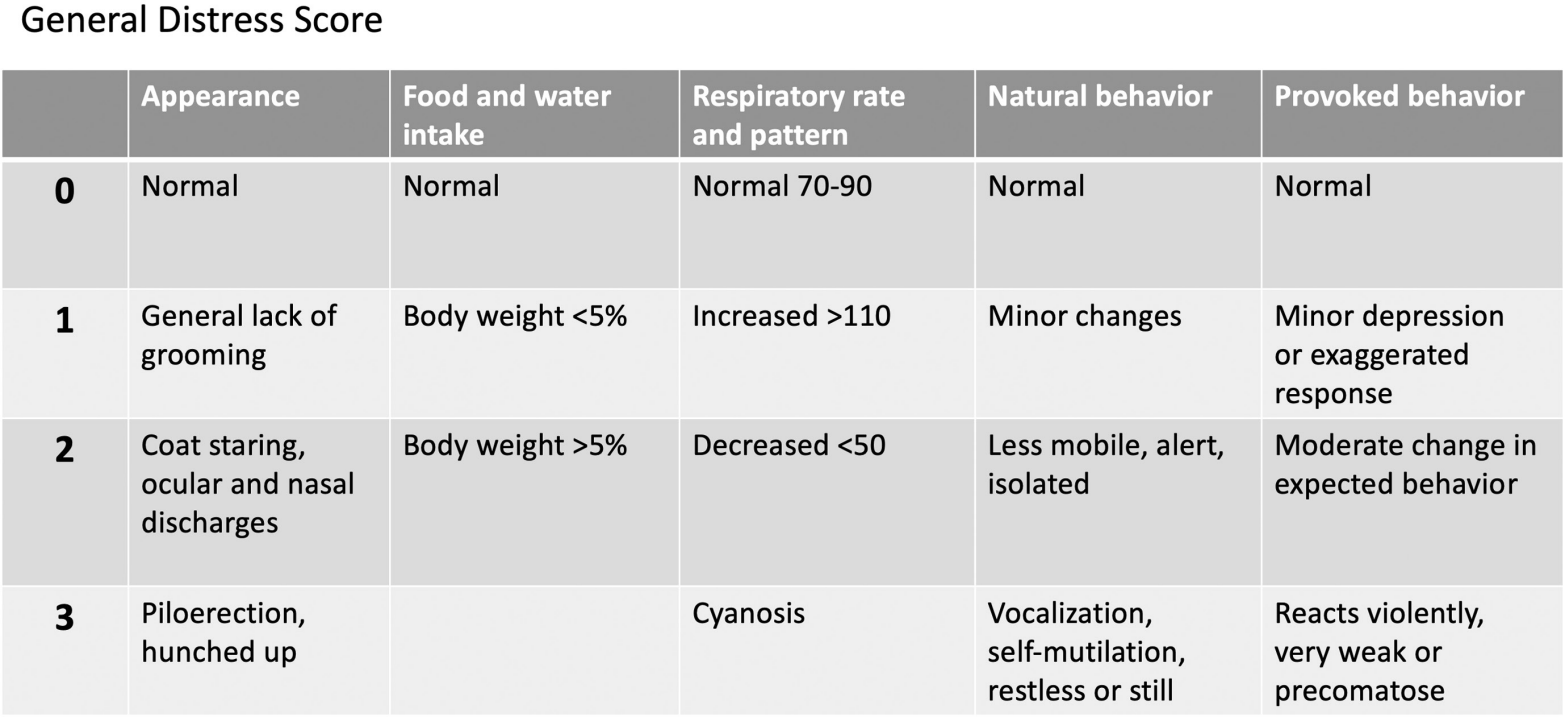
Physiological and behavioral parameters included in the GDS (general distress score). A numerical score is allocated to each categorical parameter, and a total GDS value is calculated. Animals presenting with a GDS ≥10 during the day or a GDS ≥6 at midnight were euthanized.

Animals were observed continuously for the first 3 h after surgery and subsequently scored at least four times a day using the GDS (Figure 4). The use of the GDS ensured that animal pain and distress were objectively assessed based on several physiological and behavioral parameters with a numerical score allocated to each categorical parameter: 0 for normal and 3 for completely abnormal. If 3 was scored more than once, an extra point was added for each score of 3. This ensured that severe clinical signs in one category were not outweighed by mild signs in another category. The rat grimace scale was used when assessing animal appearance.^16^ Food and water intake were evaluated by body weight, which also provided information on acute stress to the animal. The respiratory rate was measured by counting the number of breaths for 30 s and multiplying that number by 2. Our observations from the preliminary study were used when evaluating natural and provoked behaviors. Each animal was assigned a GDS value by two independent observers to see if the evaluation could be reproduced and if interobserver variation was a bias. Early euthanization was performed if the GDS was ≥10 at day. For ethical reasons, animals were also euthanized if the GDS was ≥6 at midnight.

All animals were autopsied at euthanization.

### 2.5 Blood and tissue

At 12, 24, or 48 h postsurgery, each animal was again weighed and anesthetized. Tissues were collected through the previous incision, and blood samples were collected from the heart by cannulation. Animals were euthanized by cervical dislocation under anesthesia. The liver remnant was removed, and the weight of the remaining caudate lobes was measured. PCL was fixed in phosphate‐buffered formalin for 24–48 h before being embedded in paraffin, and ACL was snap‐frozen in liquid nitrogen and then stored at −80°C until further analysis.

Blood was processed and stored at −80°C until further analysis. Alanine aminotransferase (ALT), alkaline phosphatase, haptoglobin, bilirubin, albumin, ammonia, creatinine, sodium, potassium, and phosphate were measured using a Siemens Atellica Solution Immunoassay and Clinical Chemistry analyzer (Siemens Healthcare Diagnostics Inc.). The prothrombin–proconvertin ratio (PP) was measured using a Sysmex CS‐2100i analyzer (Sysmex Corporation), an automated blood coagulation analyzer.

We evaluated liver function using the PP ratio, reflecting the initial hepatic synthetic capacity.^17^ Bilirubin was used as a marker of the liver's excretory function, haptoglobin as a marker of the synthetic function and as an acute phase reactant, and ammonia as a measure of the liver's metabolic capacity. ALT and alkaline phosphatase were used as liver injury markers.

### 2.6 Statistical analyses

We used an analysis of variance (ANOVA) to assess the differences between several groups. Head‐to‐head comparisons between groups were performed using a *t*‐test. Mean values are shown with a 95% confidence interval (CI). A *p*‐value of <0.05 was considered significant. For interobserver variation in the GDS measurements, we calculated Cohen's kappa for two raters, assigning ordinal weights. All statistical analyses were performed using Stata version 17.0 (StataCorp LLC).

## 3 RESULTS

### 3.1 Mortality and cause of death after 90% PH

During the experiment, 2 of 68 animals were excluded: 1 due to vena cava compression and 1 due to unexpected death. Further, one was euthanized shortly after surgery due to perioperative blood loss.

Fifteen animals were euthanized with a GDS ≥10 at day, and 2 animals were euthanized with a GDS ≥6 at midnight. Among animals euthanized due to a GDS ≥10, the mean survival time was 27 h after surgery (95% CI: 23–30). For animals randomized to the 48‐h group, mortality was 60%. None of the autopsies revealed identifiable causes of mortality or morbidity.

Some kind of gastric enlargement was found in all animals.

### 3.2 Comparisons of GDS values between survivors and nonsurvivors

**FIGURE 5:**
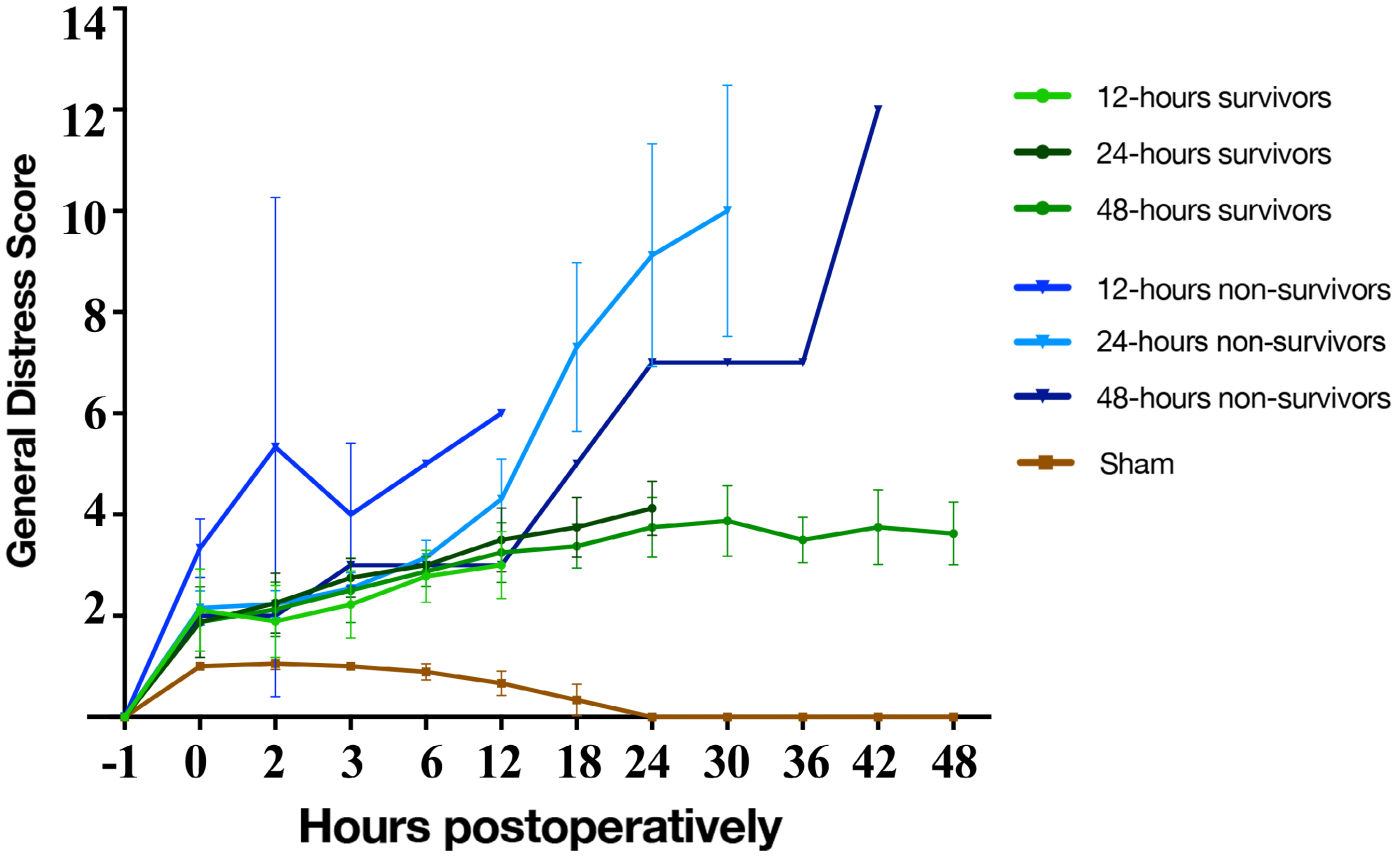
GDS (general distress score) over time. Animals were observed continuously for the first 3 h after surgery, thereafter scored at least four times a day. All scores are plotted in the graph. Early euthanization was performed if the GDS was ≥10 at day or ≥6 at midnight. Two 12‐h nonsurvivors were euthanized with a GDS ≥6 at midnight. From 18 h after PH (partial hepatectomy) and throughout the observation period, the GDS was significantly lower for 48‐h survivors compared with 24‐h nonsurvivors. Green lines: survivor subgroups, blue lines: nonsurvivor subgroups, and brown line: sham.

Except for one 12‐h nonsurvivor, the GDS increased slightly during the first 6 h after PH for all animals (Figure 5). For the 12‐h nonsurvivor, which suffered from perioperative blood loss, the GDS increased immediately after surgery. For nonsurvivors, the GDS continued to increase. From 18 h after PH and throughout the observation period, the GDS was significantly lower for 48‐h survivors compared with nonsurvivors (*p* < 0.001).

There was a near‐perfect interobserver agreement in GDS measurements (kappa: 0.98; 95% CI: 0.97–1.00).

### 3.3 Comparisons of body weight and resected liver weight between survivors and nonsurvivors

**FIGURE 6:**
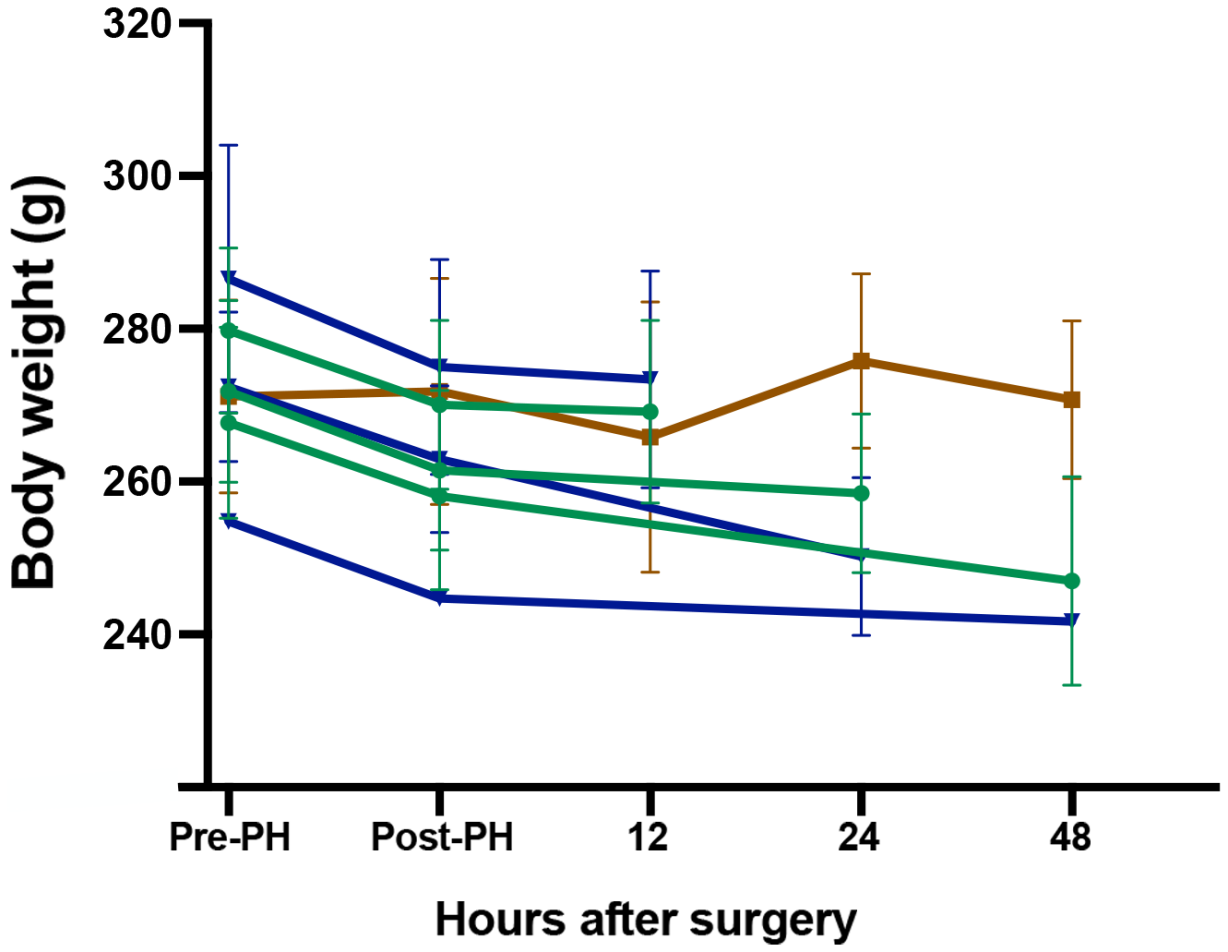
Mean body weight from presurgery to euthanization. No significant differences between survivors and nonsurvivors were found throughout the study period. Green lines: survivor subgroups, blue lines: nonsurvivor subgroups, and brown line: sham.

Body weight loss was observed for all 90% PH animals. No difference in body weight dynamics was found between survivors and nonsurvivors (Figure 6).

Mean resected liver weight was 8.6 g (95% CI: 8.2–9.0) for survivors and 8.5 g (95% CI: 8.0–9.0) for nonsurvivors.

### 3.4 Comparisons of biochemical parameters between all groups

**FIGURE 7:**
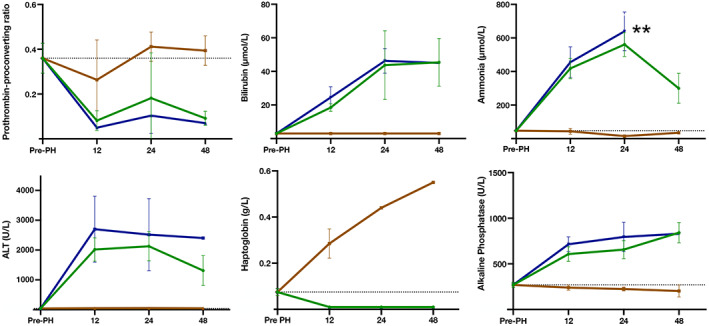
Liver‐specific biochemical parameters. *x*‐Axis: concentration, *y*‐axis: hours after surgery. Within 24 h after 90% PH (partial hepatectomy), no significant differences between survivors and nonsurvivors were found. At 48 h, decreases in ammonia and ALT (alanine aminotransferase) levels were found for survivors. Green line: survivors, blue line: nonsurvivors, brown line: sham, and gray line: baseline. **Due to a hemolyzed blood sample, no ammonia concentration is available for the 48‐h nonsurvivor.

ALT, bilirubin, alkaline phosphatase, and ammonia were elevated at all times after 90% PH (Figure 7). PP ratio decreased dramatically after resection. Haptoglobin was markedly suppressed compared to the increase observed in the sham group. No difference in any biochemical parameters was found between survivors and nonsurvivors for the first 24 h postsurgery. At 48 h, a decrease in ALT and ammonia among survivors was observed.

## 4 DISCUSSION

In the present study, we evaluated the rat model of 90% PH as a model of PHLF. We also investigated the use of a quantitative scoring system to detect lethal outcomes caused by PHLF in rats. All animals experienced PHLF after 90% PH, determined by liver‐specific biochemistry at euthanization. The nonsurvivors were probably not able to restore the minimal level of functional capacity of the liver required to avoid fatal PHLF. For survivors, PHLF appeared to be reversed over time, as indicated by a decrease in ALT and ammonia. The GDS seemed valuable in the detection of irreversible PHLF in rats, as a high GDS was associated with increased mortality.

We chose liver‐specific biochemical makers to define PHLF, as high bilirubin and low PP ratio are widely accepted as a definition of liver failure.^17^ The biochemical results did not reveal significant differences between survivors and nonsurvivors until 48 h after surgery, when ALT and ammonia decreased in survivors. Nevertheless, the clinical observations indicate a major difference in behavior between survivors and nonsurvivors already 12 h postoperatively. From this time point, the GDS started to increase for nonsurvivors. For survivors, the GDS remained almost constant from surgery to euthanization. Our extensive clinical observations showed that when the GDS reached a threshold of 6, further increase in the GDS was highly likely, ultimately leading to euthanization. The mean time for euthanization due to PHLF was 27 h postoperation. We excluded all 12‐h nonsurvivors from this calculation, because one of the animals was euthanized shortly after surgery due to perioperative blood loss, and the other two were euthanized with a GDS ≥6 at midnight. Therefore, these were not comparable to all other animals.

For all 90% PH animals, PP ratio was almost undetectably low, indicating suppressed synthetic capacity of the liver. As expected, 90% PH was followed by an increase in bilirubin, ALT, alkaline phosphatase, and ammonia. Haptoglobin was significantly depressed compared to the sham group. This is in accord with previous studies showing a 3‐day delay in haptoglobin response after 90% PH.^12^, ^18^, ^19^ The delay might be explained by the liver prioritizing its tasks during PHLF in an attempt to preserve the most vital functions. A previous regeneration study in rats by the present study group has shown that the liver function is impaired until 5 days after 90% PH, although it begins to normalize from day 3.^12^ This correlates well with the present study, only detecting the beginning of improvement in liver function for survivors, because no nonsurvivors were alive later than 43 h postoperation.

As expected, a significant mortality is associated with PHLF. The use of en bloc ligations in the rat model of 90% PH has been associated with a mortality ranging from 24% to 100%.^12^, ^13^, ^14^, ^20^ Mortality depends on not only surgical techniques but also supportive treatment where in particular glucose and testosterone have been used.^13^, ^14^, ^20^ In two different rat studies, mortality almost halved when animals were provided with 20% glucose water.^13^, ^14^ Findings regarding testosterone pretreatment and mortality are contradictory.^13^, ^20^ Additionally, no consensus on antibiotic use has been found. In the present study, neither glucose nor testosterone was administrated, because we aimed to determine the natural course of PHLF after 90% PH in rats. At the beginning of the preliminary study, antibiotics were also not administrated. However, because mortality in the preliminary study was about 95%, we introduced an injection of ampicillin before surgery. This, combined with changing the size of the ligature from 4‐0 to 5‐0, caused a decrease in mortality to 60% in the present study. The still relatively high mortality compared to the 24%^14^ and 40%^13^, ^20^ observed in other studies might be because we did not use any supportive treatment other than antibiotics. That 90% PH animals in the present study were exposed to a high level of physiological stress is further emphasized by the observed dynamics in body weight. Change in body weight is a reliable marker of acute stress in rats,^21^ and all animals exposed to 90% PH showed a rapid and pronounced decline in body weight after surgery compared to the sham group.

No previous study has systematically assessed physiological and behavioral parameters over time for rats exposed to 90% PH in an attempt to detect PHLF. However, Zieglowski et al evaluated the level of distress in rats exposed to 30% or 70% PH.^22^ The assessment of distress was based on three parameters: (1) physiological and clinical observations, (2) behavioral tests, and (3) biomarkers from blood and fecal samples. In our study, the GDS was based only on physiological and clinical parameters. Zieglowski et al. found the risk of PHLF to be at its highest 1–2 days after both 50% PH and 70% PH; the 50% PH group required 3–4 days to reach baseline evaluations compared to 5–6 days in the 70% PH group. Both the level of distress and the time needed to reach the initial clinical evaluation level can be expected to be even higher for 90% PH rats. Even though 90% PH was not performed by Zieglowski et al., the severity score seems comparable to our GDS in regard to identifying rats prone to PHLF. Based on both scoring systems, it appears that the physiological and behavioral parameters of all PH rats are maximally affected 1–2 days after liver resection regardless of the size of the liver resection. This seems to be a critical period that determines whether the rat will develop PHLF and, if so, whether it is reversible or irreversible. Additionally, our data show that physiological and clinical parameters alone are sufficient to evaluate rats exposed to PH regarding the development of irreversible PHLF. Being able to detect this without disturbing the animal is extremely valuable. This method involves no unnecessary stress to the animal that could potentially mask signs of liver failure. The present study design made it possible to capture the liver failure near the time of death but also still in time to collect samples from the animal.

Our study has several strengths. First, we conducted a pilot study of 60 animals before the present study to standardize the procedure, and all operations were performed by one surgeon. Second, a near‐perfect interobserver agreement for GDS evaluations ensures reliability of this parameter. Third, we randomized the animals to the different study arms to ensure that bias would not be introduced due to potential variances between litters. Limitations include the lack of observation of animals during the night. For ethical reasons, we decided to euthanize animals eliciting a GDS of ≥6 at midnight. Only two animals were euthanized for this reason, and only one rat died unexpectedly overnight. Thus, the lack of observations at night is of limited concern.

Laboratory rats serve as an accelerated model for the study of liver regeneration in mammals. However, results obtained in rat liver models cannot necessarily be directly transferred to humans because of the differences between species regarding anatomy, liver regeneration kinetics, and clinical complications after surgery. Nonetheless, being able to execute controlled rat liver studies of the minimal‐size FLR allows us to gain important basic knowledge concerning PHLF and liver regeneration.

In conclusion, based on our clinical scoring system and supported by liver‐specific biochemistry, the model of 90% PH seems to be relevant for investigating PHLF in rats. By including both physiological and behavioral parameters, the GDS appears to be valuable in detecting lethal outcomes caused by PHLF in rats.

## AUTHOR CONTRIBUTIONS

Study conception and design: Andrea Lund, Marie Ingemann Pedersen, Kasper Jarlhelt Andersen, Michelle Meier, Anders Riegels Knudsen, and Frank Viborg Mortensen. Data collection: Andrea Lund and Marie Ingemann Pedersen. Data analysis and interpretation: Andrea Lund, Jakob Kirkegård, Michelle Meier, Anders Riegels Knudsen, and Frank Viborg Mortensen. Drafting of the manuscript: Andrea Lund, Jakob Kirkegård, and Frank Viborg Mortensen. Critical revision of the manuscript: Andrea Lund, Jakob Kirkegård, Anders Riegels Knudsen, Michelle Meier, Marie Ingemann Pedersen, Kasper Jarlhelt Andersen, and Frank Viborg Mortensen.

## CONFLICT OF INTEREST STATEMENT

None. The funding sources did not have any roles in investigation, data interpretation, or paper presentation.

## ETHICS STATEMENT

All procedures were conducted in accordance with the Danish Animal Research Commitee, Copenhagen, Denmark, and under the authority of the Project Licence (license number: 2021‐15‐0201‐00978). Animals received care in accordance with the Guide for the Care and Use of Laboratory Animals published by the U.S. National Institutes of Health.^23^
